# Vertigo: Giddiness in Relation to General Medicine

**Published:** 1912-05-11

**Authors:** 


### VERTIGO.

### Giddiness in Relation to General Medicine.

Vertigo literally means a "turning" or giddiness. It is a condition which is characterised by a feeling of swimming in the head, oscillation of the body, and rotation of external objects, which sensations may be associated with a tendency to reel or stagger. It is defined by Stephen Mackenzie as "the consciousness of disordered equilibration." It is a disorder of the functions by which the equilibrium of the body is maintained. There are two forms: (*a*) subjective; (*b*) objective. In the former there is an apparent movement of the patient himself; in the latter there is an apparent movement of external objects. There are many sensations associated with vertigo, *e.g.* a tendency to incline to the right or left, to turn to the right or left, to fall backwards or forwards, a feeling of sinking downwards or of falling from a height, etc.

It is a distressing symptom which may be due to many causes, some trifling, some serious, and so requires very careful investigation in order that an accurate diagnosis may be made.

### Aural or Labyrinthine Vertigo.

This form of vertigo usually occurs in severe paroxysms and is associated with tinnitus and deafness. It rarely affects people under thirty years of age, and is commoner in men than in women. It is due to disease of the labyrinth and the nerve endings which it contains.

The chief causes are: (1) Inflammatory disease of the labyrinth—rheumatic, gouty, syphilitic; (2) Hæmorrhage into the labyrinth, *e.g.* in leuchæmia; (3) Degenerative changes in the membrane (senile); (4) Organic disease of the vestibular nerve; (5) Increased labyrinthine pressure—(*a*) otitis media; (*b*) cerumen blocking external auditory meatus; (*c*) violent syringing of the ears; (*d*) obstruction of the Eustachian tube; (*e*) spasm of the tensor tympani muscle; (*f*) paralysis of the stapedius.

The attacks are characterised by the sudden onset of giddiness, which may occur spontaneously or be excited by some sudden movement such as coughing or sneezing. The paroxysm may be so severe that the patient falls to the ground and for a moment there may be a loss of consciousness. There may be only a tendency to fall or turn, or there may be a sensation as if external objects are moving, or both these sensations may be associated. Tinnitus and deafness are also present in the majority of cases. The deafness varies in intensity—it may be bilateral or unilateral, generally the former.

After the vertigo has lasted for a short time there is usually a feeling of nausea, which is followed by vomiting, and this is succeeded by pallor and the occurrence of a cold clammy sweat which breaks out on the skin. In some cases slight nystagmus and diplopia may be noticed during the paroxysm. The diagnosis of aural vertigo, therefore, is based on the association of paroxysms of vertigo with tinnitus and deafness due to morbid changes disturbing the functions of the labyrinth.

### Organic Disease of the Brain.

Vertigo is one of the most constant symptoms of cerebellar disease, and its character must be carefully investigated, as it may help to determine whether the tumour is within or external to the cerebellum. The determination of this point is of the greatest practical importance as it is possible to remove the tumour should it be extra-cerebellar. In both varieties of tumour the objective vertigo is from the side of the lesion to the healthy side, *e.g.* with a tumour on the right side the apparent movement of external objects is from right to left. In intra-cerebellar tumour the subjective vertigo is also from the side of the lesion to the healthy side, but in extra-cerebellar tumour it is in the opposite direction. Vertigo from this cause is associated with other symptoms: headache, pain in the occipital region and back of the neck, vomiting, optic neuritis, nystagmus, ataxy of the limbs, irregular staggering gait like that of a drunken man, paresis of the trunk muscles so that the patient can neither bend his trunk nor raise it when lying in bed without the aid of his arms, and frequently convergent strabismus from sixth nerve paralysis.

Vertigo is one of the classical symptoms of cerebral as well as of cerebella tumour. The system—complex—would be: headache, vomiting, optic neuritis, and vertigo. It is liable to occur if the patient turns round quickly or suddenly rises from the supine to the erect posture.

Vertigo may be one of the premonitory symptoms of cerebral hæmorrhage.

### Epilepsy.

A sudden attack of vertigo may constitute the *aura* of major epilepsy or may be the only manifestation of a mild attack of the disease. It is usually subjective; the patient feels that he is turning round rather than that external objects are moving. The diagnosis is obvious if it is followed by sudden loss of consciousness and tonic and clonic convulsions during which there is frothing at the mouth, the tongue being bitten and urine and fæces passed unconsciously. Even in the mild attacks there is loss of consciousness of varying length followed by mental dulness, and this is the most distinguishing feature of vertigo due to this cause.

Giddiness is frequently one of the prominent symptoms of nervous exhaustion from over-work, mental anxiety, sexual excesses, and other causes. It is also common in traumatic neurasthenia. It is independent of any aural disease, and is never so severe as true aural vertigo. As a rule it is only felt when the patient is in the erect position and is liable to occur on suddenly raising the head. It may be associated with insomnia, flatulence, nausea, and palpitation.

### Migraine.

Vertigo may be one of the symptoms of migraine. As a rule it is not severe and is associated neither with tinnitus nor deafness. It usually follows the disorders of sight and accompanies the headache which occurs in this disease. Sometimes vertigo replaces the typical attack of migraine. The association of giddiness with intense paroxysmal unilateral headache, transient hemianopia, flashes of light, the production of zigzag patterns, and other disorders of sight, the curious spasmodic contraction and dilation of the pupils termed hippus and numbness of the tongue and face would point without much doubt to a diagnosis of migraine.

### Ocular Vertigo.

Vertigo is frequently associated with oculo-motor paralysis and is due to the diplopia and the erroneous impressions which, in consequence, are produced as to the exact position of external objects, *i.e.* to what is termed erroneous projection or the false conception of the position of external objects. This form of vertigo is seldom intense, and only occurs when the paralysed muscle is in action. On closing the paralysed eye the feeling of vertigo at once disappears. It may occur with paralysis of a single muscle, such as the external rectus, or may be associated with insufficiency of the internal recti muscles, a condition which is termed muscular asthenopia and is most frequently due to myopia, but may occur as a result of other errors of refraction, and also as a sequela of exhausting diseases such as typhoid and diphtheria. When due to muscular asthenopia it usually comes on after reading. It is produced by the internal recti becoming exhausted from the prolonged strain exerted to maintain convergence of the eyes in order to focus the letters and to these muscles suddenly giving way; in consequence of this the eyeballs diverge, the letters become indistinct, and a feeling of giddiness is produced. In addition to vertigo the patient usually complains of headache, nausea, and aching at the back of the eyes. It is, therefore, of the greatest importance in a case of vertigo, especially if it is brought on by reading, carefully to investigate the condition of the eyes, and to correct any errors of refraction which may be present.

### Cardio-Vascular.

Vertigo is a common symptom of disease of the heart and of the blood vessels. It may be one of the earliest signs of aortic disease, the patient noticing it on suddenly rising from the supine to the erect posture as when first getting up in the morning or on raising the head after bending downwards as in the attitude for lacing the boots. The presence of other signs and symptoms of aortic disease would point to the correct diagnosis of its cause, *e.g.* anæmia, visible and forcible pulsation of the carotids, the characteristic splashing pulse of aortic regurgitation or the slow and continued pulse of aortic stenosis, the physical signs of enlargement of the heart, the early diastolic bruit of aortic regurgitation and the systolic thrill and bruit over the second right intercostal space of aortic stenosis. Vertigo may be associated with other forms of valvular disease and also with the syncopal attacks that accompany degenerative changes in the myocardium. It is also a frequent symptom of arterio-sclerosis, and when associated with a slow pulse, syncopal or epileptiform attacks, constitutes the syndrome termed Stokes-Adams disease. The arterio-sclerosis may or may not be associated with chronic Bright's disease.

Giddiness may also be produced by sudden alterations in the cerebral circulation, *e.g.* by either anæmia or hyperanæmia of the brain. It is very liable to occur in women at the climacteric and to be associated with other signs of vasomotor disturbances, such as local sweating, hot flushings, etc.

### Gastric Vertigo.

This form of vertigo occurs most frequently in persons of middle age who suffer from dyspepsia. It is accompanied by neither tinnitus nor deafness, but as a rule with some of the following symptoms: epigastric pain, a feeling of distension in the upper part of the abdomen, heartburn, flatulence, palpitation, nausea, vomiting, and constipation. The attacks may come on suddenly and be followed by headache, pallor, and a feeling of coldness in the extremities.

Gowers states that "Vertigo of purely gastric origin does not constitute more than 5 per cent. of the cases in which definite giddiness is the prominent symptom." He believes the condition is the result of "unobtrusive" labyrinthine disease.

### Nasal and Laryngeal.

Vertigo may be a symptom of nasal disease, especially of nasal obstruction from the presence of polypi. The chief evidence that it is due to such a cause is the relief which follows the cure of the nasal disease. When investigating, therefore, the cause of vertigo a careful examination of the nares should be made.

This form is associated with the presence of spasmodic cough and signs of laryngeal irritation. The cough usually precedes the feeling of vertigo, and appears to be the cause of it, probably by producing cerebral congestion.

### Other Forms.

The administration of certain drugs, especially quinine and the salicylates, may give rise to well-marked vertigo. They probably act on the labyrinth through the vascular system, or possibly on the auditory or cortical centres which have to do with the consciousness of auditory perceptions.

Vertigo may also be caused by the excessive use of alcohol, tobacco, or tea.

In the nocturnal form a feeling of giddiness or a sensation of falling from a height is experienced by the patient after retiring to bed and just before falling asleep.

Gowers considers it to be a mild form of labyrinthine vertigo, which is due to a sudden spasm of the tympanic muscle, leading to a change of pressure within the labyrinth. That it is due to contraction of an aural muscle is suggested by the fact that many affected in this way are conscious of the curious vibratory sound which is characteristic of the contraction of an intra-aural muscle.

